# Supplementary material for: Two Non-Necrotic Disease Resistance Types Distinctly Affect the Expression of Key Pathogenic Determinants of Xanthomonas euvesicatoria in Pepper
Source: Plants (Basel). 2022 Dec 24;12(1):89. doi: 10.3390/plants12010089 (PMC9824575; doi:10.3390/plants12010089)
Supplement: Supplementary file 1 [file plants-12-00089-s001.zip › plants-1978627-supplementary.pdf]

Figure S1. Confirmation of the identity of *Xanthomonas euvesicatoria* SZJ01 with specific PCR reaction.

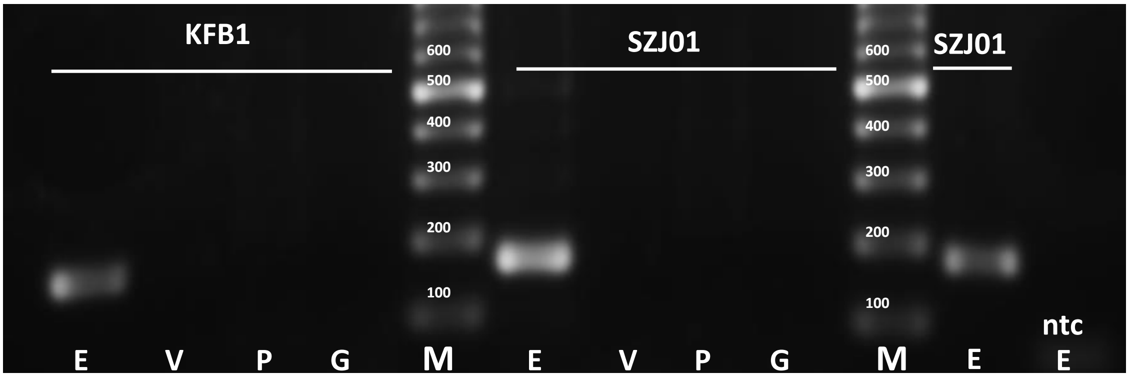

Xanthomonas euvesicatoria SZJ01 strain was isolated from pepper leaves in Hungary (by János Szarka). The identity of this *Xanthomonas* was determined by PCR according to Araújo et al. 2012 [32]. Specific primer pairs were used for PCR reactions: **E**, *Xanthomonas euvesicatoria*, **V**, *Xanthomonas vesicatoria*, **P**, *Xanthomonas perforans*, **G**, *Xanthomonas gardneri*. Only the PCR reaction with X. *euvesicatoria*-specific primer pair resulted in a specific product (173 bp). *Xanthomonas euvesicatoria* KFB1 strain were used as a reference strain in PCR reactions [33]. The amplicons were separated on agarose gels (1.5 %) by electrophoresis in 1X TBE buffer. **M**, GeneRuler 100 bp Plus DNA Ladder was used as a molecular marker (Thermo Scientific). **ntc E**, no template control of PCR reaction that was carried out with *Xanthomonas euvesicatoria* specific primers.
